# Clinical significance of concomitant bacteriuria in patients with *Staphylococcus aureus* bacteraemia

**DOI:** 10.1007/s10096-023-04559-z

**Published:** 2023-02-02

**Authors:** Matthaios Papadimitriou-Olivgeris, Damien Jacot, Laurence Senn, Benoit Guery

**Affiliations:** 1 Infection Prevention and Control Unit, Lausanne University Hospital and University of Lausanne, Lausanne, Switzerland; 2 Infectious Diseases Service, Lausanne University Hospital and University of Lausanne, 1011 Lausanne, Switzerland; 3 Institute of Microbiology, Lausanne University Hospital and University of Lausanne, Lausanne, Switzerland

**Keywords:** *Staphylococcus aureus*, Bloodstream infection, Bacteraemia, Bacteriuria, Infective endocarditis

## Abstract

This retrospective study, conducted at Lausanne University Hospital (2015–2021), compared *Staphylococcus aureus* bacteraemia (SABA) patients with or without concomitant bacteriuria (SABU). Among 448 included bacteraemic patients, 62 (13.8%) had *S. aureus* concurrently isolated from urine. In multivariate analysis, there was a significant difference in the odds of community-onset bacteraemia (*P* 0.030), malignancy (*P* 0.002), > 1 pair of positive blood cultures (*P* 0.037), and persistent bacteraemia for at least 48 h (*P* 0.045) in patients with concurrent SABU. No difference concerning mortality was found. On the other hand, SABU was associated with higher rates of SABA recurrence after antibiotic cessation.

**Supplementary Information:**

The online version contains supplementary material available at 10.1007/s10096-023-04559-z.

## Introduction

*Staphylococcus aureus* bacteraemia (SABA) remains a frequent infection that is associated with increased mortality [1]. SABA is commonly complicated by secondary foci of infection such as endocarditis, osteoarticular infections, and infection of prosthetic material [2–4]. *S. aureus* isolation from urine cultures is a rare occurrence and usually represents asymptomatic bacteriuria, primary urinary tract infection especially in patients with indwelling urinary tract devices or hematogenous seeding of the urinary tract. In case of SABA, presence of concomitant bacteriuria (SABU) has been associated with presence of endocarditis or bone and joint infection and was a predictor of complicated bacteraemia or mortality [2, 5, 6].

The aim of the present study was to determine the clinical relevance of concomitant bacteriuria in patients with SABA analyzing the patients’ profile and prognosis.

## Materials and methods

This retrospective study was conducted at the Lausanne University Hospital, Lausanne, Switzerland. The study was approved by the ethic committee of the Canton of Vaud (CER-VD 2021–02,516).

Inclusion criteria were adult patients (≥ 18 years old) with SABA and a urine culture within 48 h of SABA onset between January 1st, 2015 and December 31st, 2021. Exclusion criteria were patients’ prior written refusal of use of their data, a urinary tract infection as the source of the bacteraemia, and a urine culture collected from a patient already on antimicrobial treatment.

Blood cultures were incubated with the BACTEC™ FX blood culture system (Becton Dickinson, USA). Urinary cultures were inoculated on CHROMagar Orientation Medium plates (Becton Dickinson) with a zig-zag streaking pattern for quantification and incubated 18 h prior identification. Matrix-assisted laser desorption-ionization time of flight mass spectrometry (MALDI-TOF MS, Bruker Daltonics, Bremen, Germany) was used for the identification to the species level. Susceptibility results were obtained using the Vitek® 2 (BioMérieux, Marcy-l’Étoile, France) and were evaluated according the EUCAST criteria [7].

Data regarding demographics (age, sex), comorbidities, symptoms, signs, laboratory results (white blood cells, platelets, C-reactive protein, procalcitonin), presence of sepsis or septic shock, and type of infection were collected from patients’ electronic health records. A secondary analysis was performed by comparing patients with urine culture performed and those without.

The date of collection of the first positive blood culture was defined as infection onset. Infection was categorized as sepsis or septic shock according to definition proposed by the Third International Consensus [8].

SPSS version 26.0 (SPSS, Chicago, IL, USA) software was used for data analysis. Categorical variables were analyzed using the chi-square or Fisher exact test and continuous variables with Mann–Whitney *U* test. Covariates were tested for multi-collinearity through variance inflation factor assessment; those not collinear and clinically relevant were used in multivariate analysis. A multivariate logistic regression analysis was performed having SABU as the dependent variable. Odds ratios (ORs) and 95% confidence intervals (CIs) were calculated to evaluate the strength of any association. All statistic tests were 2-tailed and *P* < 0.05 was considered statistically significant.

## Results

**Fig. 1:**
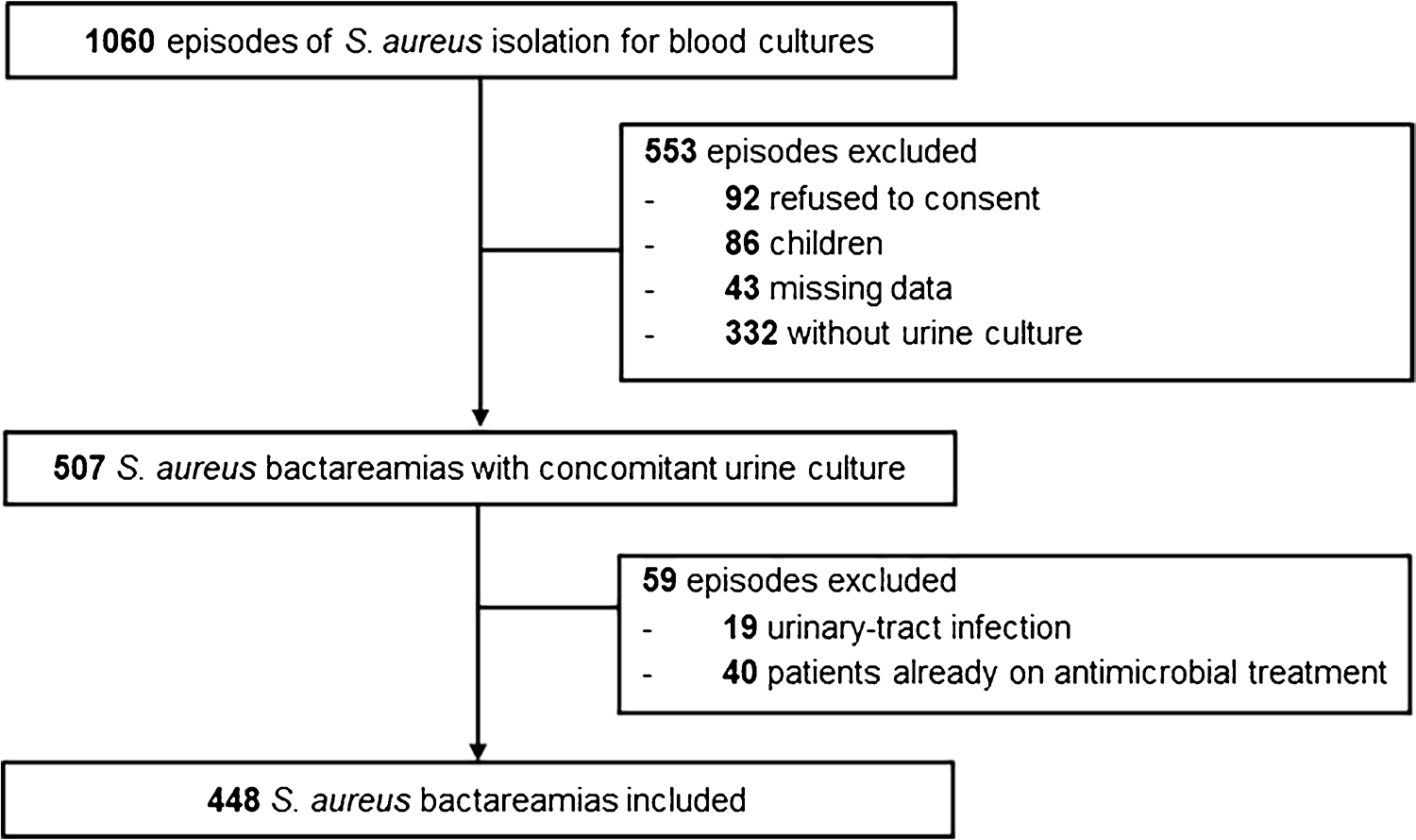
Flowchart of included patients

Among 1060 episodes of SABA, we identified 467 episodes with a concomitant urine culture that met the inclusion criteria (Fig. 1). Nineteen episodes were excluded since they were attributed to primary urinary tract infection (all patients had urinary tract prosthetic material), resulting in 448 episodes included in the analysis. Thirty-four bacteraemic isolates (7.5%) were methicillin-resistant. Most common types of infection were osteoarticular (133; 29.7%), bacteraemia of unknown origin (104; 23.2%), and proven endocarditis (58; 12.9%). Sixty-two (13.8%) episodes had *S. aureus* concurrently isolated from urine. Demographic and clinical characteristics of patients with concomitant bacteriuria and those without are shown in Table 1. Site of infection and outcomes of SABA episodes are shown in Supplementary Tables 1 and 2, respectively. SABU rate was higher among patients with bone and joint infection and especially native osteoarticular infection, embolic events, and persistent bacteraemia. Patients with SABU had higher rates of SABA recurrence after antibiotic cessation. In multivariate analysis, there was a significant difference in the odds of community-onset bacteraemia (OR 1.43, CI 1.04–1.96;* P* 0.030), malignancy (OR 2.80, CI 1.47–1.96;* P* 0.002), > 1 pair of positive blood cultures (OR 1.78, CI 1.04–3.06;* P* 0.037), and persistent bacteraemia for at least 72 h (OR 1.24, CI 1.00–1.52;* P* 0.045) in patients with concurrent SABU (Table 1). When comparing the 448 episodes included in the study and the 332 episodes that did not have urine culture performed (Supplementary Table 3), urine culture was more commonly performed in patients that had community-acquired SABA, with no obvious site of infection and more severely ill patients.

**Table 1:** Demographics and clinical characteristics of *S. aureus* bacteraemic patients with and without concomitant bacteriuria

	Univariate analysis					Multivariate analysis	
	Without SABU (*n* = 386)		With SABU (*n* = 62)		*P*	OR (95% CI)	*P*
Demographics							
Male sex	261	67.6%	51	82.3%	0.020		
Age (years)	70	56–79	68	54–78	0.343		
Comorbidities							
Diabetes mellitus	112	29.0%	19	30.6%	0.793		
Chronic kidney disease (moderate or severe)	100	25.9%	8	12.9%	0.026		
Malignancy (solid organ or hematologic)	77	19.9%	21	33.9%	0.014	2.80 (1.47–1.96)	0.002
Obesity	104	26.9%	18	29.0%	0.732		
Immunosuppression	82	21.2%	8	12.9%	0.128		
Location of infection onset							
Community	206	53.4%	44	71.0%	0.010^a^	1.43 (1.04–1.96)	0.030
Hospital	180	46.6%	18	29.0%			
Cardiac predisposing factors (according to modified Duke criteria)	53	13.7%	9	14.5%	0.868		
Urinary catheter	32	8.3%	6	9.7%	0.716		
Microbiological data							
> 1 pair of positive blood cultures	298	77.2%	58	93.5%	0.003	1.78 (1.04–3.06)	0.037
Polymicrobial bloodstream infection	40	10.4%	2	3.2%	0.074		
Methicillin-resistance	29	7.5%	5	8.1%	0.799		
Time to blood culture positivity (h)	12	9–16	12	9–14	0.368		
Duration of bacteraemia ≥ 48 h	92	23.8%	28	45.2%	< 0.001	1.24 (1.00–1.52)	0.045
Infection data							
Fever	324	83.9%	53	85.5%	0.757		
Duration of general symptoms (days)	1	1–2	2	1–4	< 0.001		
Heart murmur	115	29.8%	27	43.5%	0.031		
Embolic events	46	11.9%	13	21.0%	0.050	1.22 (0.67–2.60)	0.615
Sepsis	184	47.7%	30	48.4%	0.916		
Main infection sites							
Unknown origin	92	23.8%	12	19.4%	0.438		
Osteoarticular	108	28.0%	25	40.3%	0.014		
Native osteoarticular (septic arthritis, vertebral and acute or chronic non-vertebral osteomyelitis)	82	21.2%	22	35.5%	0.014	1.27 (0.54–2.99)	0.590
Prosthetic osteoarticular (prosthetic joint, osteosynthesis or spondylodesis infection)	31	8.0%	3	4.8%	0.604		
Proven endocarditis (including CIED cable infection)	48	12.4%	10	16.1%	0.424		
Complicated bacteraemia	201	52.1%	43	69.4%	0.011		

Data are depicted as number and percentage or median and Q1–3

^a^Comparison between community and hospital-acquired infection

## Discussion

In the present study, SABU was detected among 13.8% of patients with SABA and performed urine culture, rate comparable to the literature (10.5–33.8%) [2, 5, 6, 9–11]. In contrast to other studies, no association between SABU and endocarditis was observed [1, 5, 6]. In the present study, presence of SABU was associated with native osteoarticular infection. As previously shown, vertebral osteomyelitis and complicated SABA in general were associated with SABU [2, 9, 10]. In a meta-analysis, bone and joint infections were more common in patients with SABU, even though in total only 45 patients had such a complication in the meta-analysis as compared to 104 included in the present study [1].

As previously shown [2, 9], SABU was more common in community-acquired bacteraemia, probably since such patients have longer duration of bacteraemia before first blood culture collection as compared to nosocomial cases. In the present study, both multiple initial positive blood cultures and duration of bacteraemia after first blood culture collection were independently associated with SABU. Among aforementioned factors, only persistent bacteraemia was previously found to be associated with SABU [5, 10]. Both factors outline the high bacterial load of the infection which in turn could lead to transcytosis though endothelial cells, as found in murine models [12].

As previously discussed, the higher burden of bacteraemia could also explain the reported higher rates of SABA recurrence in SABU patients [5, 10]. Thus, clinicians, and subsequently patients with SABU, should be attentive of an increased risk of SABA recurrence after antibiotic cessation. SABU was considered as a surrogate of complicated course of infection, being associated with higher rates of septic shock, ICU admission, ICU mortality, and SABA recurrence [6, 9–11]. In the present study with the exception of SABA recurrence, no other association was found.

This study has several limitations. It is a single-center retrospective study and urine samples were not obtained in all patients with SABA. Despite that, to the best of our knowledge, the present study included the largest population to date.

In conclusion, no impact of SABU on severity of disease or mortality was found in the present study. Presence of concomitant bacteriuria in patients with SABA may be a useful predictor of persistent bacteraemia and SABA recurrence.

## Supplementary Information

Below is the link to the electronic supplementary material.

Supplementary file1 (DOCX 31 KB)

## Data Availability

The datasets generated during and/or analyzed during the current study are not publicly available, but are available from the corresponding author on reasonable request.
