# Supplementary material for: Clinical significance of concomitant bacteriuria in patients with Staphylococcus aureus bacteraemia
Source: Eur J Clin Microbiol Infect Dis. 2023 Feb 2;42(3):379–82. doi: 10.1007/s10096-023-04559-z (PMC9899163; doi:10.1007/s10096-023-04559-z)
Supplement: Supplementary file 1 — Supplementary file1 (DOCX 31 KB) [file 10096_2023_4559_MOESM1_ESM.docx]

**Supplementary Table 1:** Infection sites of *S. aureus* bacteraemic patients with and without concomitant bacteriuria

|  | Without SABU (n=386) | | With SABU  (n=62) | | *P* |
| --- | --- | --- | --- | --- | --- |
| Unknown origin | 92 | 23.8% | 12 | 19.4% | 0.438 |
| Central venous catheter-related | 48 | 12.4% | 5 | 8.1% | 0.401 |
| Peripheral venous catheter-related | 18 | 4.7% | 3 | 4.8% | 1.000 |
| Lower-respiratory tract | 44 | 11.4% | 4 | 6.5% | 0.374 |
| Skin and soft tissue | 22 | 5.7% | 3 | 4.8% | 1.000 |
| Osteoarticular | 108 | 28.0% | 25 | 40.3% | 0.014 |
| Native osteoarticular | 82 | 21.2% | 22 | 35.5% | 0.014 |
| Septic arthritis | 39 | 10.1% | 12 | 19.4% | 0.033 |
| Acute non-vertebral osteomyelitis | 41 | 10.6% | 8 | 12.9% | 0.593 |
| Chronic non-vertebral osteomyelitis | 35 | 9.1% | 6 | 9.7% | 0.877 |
| Vertebral osteomyelitis | 32 | 8.3% | 10 | 16.1% | 0.049 |
| Prosthetic osteoarticular with prosthesis | 31 | 8.0% | 3 | 4.8% | 0.604 |
| Prosthetic joint infection | 23 | 6.0% | 3 | 4.8% | 1.000 |
| Osteosynthesis infection | 5 | 1.3% | 0 | 0% | 1.000 |
| Spondylodesis infection | 3 | 0.8% | 0 | 0% | 1.000 |
| Proven endocarditis (including CIED cable infection) | 48 | 12.4% | 10 | 16.1% | 0.424 |
| Other | 29 | 7.5% | 6 | 9.7% | 0.556 |

Data are depicted as number and percentage or median and Q1-3

**Supplementary Table 2:** Outcomes of *S. aureus* bacteraemic patients with and without concomitant bacteriuria

|  | Without SABU (n=386) | | With SABU  (n=62) | | *P* |
| --- | --- | --- | --- | --- | --- |
| In-hospital mortality | 83 | 21.5% | 11 | 17.7% | 0.500 |
| Mortality at 14d | 41 | 10.6% | 8 | 12.9% | 0.593 |
| Mortality at 28d | 58 | 15.0% | 10 | 16.1% | 0.822 |
| Mortality at 120d | 87 | 22.5% | 14 | 22.6% | 0.994 |
| Mortality at 1y (among 323 patients) | 101 | 35.9% | 20 | 47.6% | 0.145 |
| Recurrence of bacteremia at 120d from antibiotic termination | 9 | 2.3% | 5 | 8.1% | 0.032 |

Data are depicted as number and percentage or median and Q1-3

**Supplementary Table 3:** Demographics and clinical characteristics of patients for which urine culture was performed as compared to those that it was not

|  | No urine culture (n=332) | | Urine culture  (n=448) | | *P* |
| --- | --- | --- | --- | --- | --- |
| Demographics |  |  |  |  |  |
| Male sex | 240 | 72.3% | 312 | 69.6% | 0.422 |
| Age (years) | 64 | 53-76 | 70 | 56-80 | 0.001 |
| Co-morbidities |  |  |  |  |  |
| Congestive heart failure | 17 | 5.1% | 39 | 8.7% | 0.055 |
| Chronic obstructive pulmonary disease | 40 | 12.0% | 53 | 11.8% | 1.000 |
| Cirrhosis | 25 | 7.5% | 49 | 10.9% | 0.108 |
| Diabetes mellitus | 103 | 31.0% | 131 | 29.2% | 0.591 |
| Chronic kidney disease (moderate or severe) | 72 | 21.7% | 108 | 24.1% | 0.428 |
| Malignancy (solid organ or haematologic) | 49 | 14.8% | 98 | 21.9% | 0.012 |
| Obesity | 70 | 21.1% | 122 | 27.2% | 0.049 |
| Immunosuppression | 47 | 14.2% | 90 | 20.1% | 0.031 |
| Charlson Comorbidity Index | 5 | 2-7 | 5 | 3-8 | 0.001 |
| Location of infection onset |  |  |  |  |  |
| Community | 242 | 72.9% | 250 | 55.8% | <0.001^a^ |
| Hospital | 90 | 27.1% | 198 | 44.2% |  |
| Cardiac predisposing factors (according to modified Duke criteria) | 67 | 20.2% | 62 | 13.8% | 0.018 |
| Presence of prosthetic material (excluding cardiac valve) |  |  |  |  |  |
| Cardiac implantable electronic devices | 30 | 9.0% | 51 | 11.4% | 0.288 |
| Endovascular (non-cardiac) prosthetic material | 20 | 6.0% | 21 | 4.7% | 0.408 |
| Osteoarticular prosthetic material | 87 | 26.2% | 100 | 22.3% | 0.235 |
| Microbiological data |  |  |  |  |  |
| >1 pair of positive blood cultures | 250 | 75.3% | 356 | 79.5% | 0.167 |
| Polymicrobial bloodstream infection | 29 | 8.7% | 42 | 9.4% | 0.759 |
| Methicillin-resistance | 24 | 7.2% | 34 | 7.6% | 0.850 |
| Time to blood culture positivity (h) | 3 | 10-17 | 12 | 9-16 | 0.045 |
| Time to blood culture positivity ≤13h | 173 | 52.1% | 259 | 57.8% | 0.113 |
| Duration of bacteraemia (h) | 0 | 0-45 | 0 | 0-53 | 0.115 |
| ≥48h | 78 | 23.5% | 120 | 26.8% | 0.296 |
| Infection data |  |  |  |  |  |
| Fever | 276 | 83.1% | 377 | 84.2% | 0.703 |
| Duration of general symptoms (days) | 1 | 1-2 | 2 | 1-2 | 0.122 |
| Heart murmur | 94 | 28.3% | 142 | 31.7% | 0.309 |
| Embolic events | 56 | 16.9% | 59 | 13.2% | 0.150 |
| Sepsis | 117 | 35.2% | 214 | 47.8% | <0.001 |
| Septic shock | 40 | 12.0% | 81 | 18.1% | 0.021 |
| SOFA score | 2 | 0-4 | 3 | 1-5 | <0.001 |
| Infection site |  |  |  |  |  |
| Unknown origin | 51 | 15.4% | 104 | 23.2% | 0.007 |
| Central venous catheter-related | 22 | 6.6% | 53 | 11.8% | 0.015 |
| Peripheral venous catheter-related | 13 | 3.9% | 21 | 4.7% | 0.602 |
| Lower-respiratory tract | 28 | 8.4% | 48 | 10.7% | 0.288 |
| Skin and soft tissue | 36 | 10.8% | 25 | 5.6% | 0.007 |
| Osteoarticular | 123 | 37.0% | 133 | 29.7% | 0.030 |
| Native osteoarticular | 78 | 23.5% | 104 | 23.2% | 0.927 |
| Septic arthritis | 31 | 9.3% | 51 | 11.4% | 0.356 |
| Acute non-vertebral osteomyelitis | 53 | 16.0% | 49 | 10.9% | 0.040 |
| Chronic non-vertebral osteomyelitis | 42 | 12.7% | 41 | 9.2% | 0.117 |
| Vertebral osteomyelitis | 21 | 6.3% | 42 | 9.4% | 0.122 |
| Prosthetic osteoarticular with prosthesis | 47 | 14.2% | 34 | 7.6% | 0.003 |
| Prosthetic joint infection | 29 | 8.7% | 26 | 5.8% | 0.114 |
| Osteosynthesis infection | 11 | 3.3% | 5 | 1.1% | 0.032 |
| Spondylodesis infection | 5 | 1.5% | 3 | 0.7% | 0.296 |
| Proven endocarditis (including CIED cable infection) | 48 | 14.5% | 58 | 12.9% | 0.543 |
| Modified Duke imaging criterion | 35 | 10.5% | 42 | 9.4% | 0.590 |
| Other | 33 | 9.9% | 35 | 7.8% | 0.298 |
| Complicated bacteraemia | 206 | 62.0% | 244 | 54.5% | 0.034 |
| Laboratory data |  |  |  |  |  |
| White blood cells (×10^9^/l) | 12.1 | 8.9-16.2 | 12.4 | 8.3-16.6 | 0.946 |
| Platelets (×10^9^/l) | 218 | 1144-305 | 208 | 120-304 | 0.127 |
| C-reactive protein (mg/l) (among (724 patients) | 194 | 101-301 | 206 | 105-302 | 0.863 |
| Outcome |  |  |  |  |  |
| In-hospital mortality | 57 | 17.2% | 94 | 21.0% | 0.183 |
| Mortality at 14d | 30 | 9.0% | 49 | 10.9% | 0.384 |
| Mortality at 28d | 47 | 14.2% | 68 | 15.2% | 0.691 |
| Mortality at 120d | 63 | 22.5% | 101 | 26.7% | 0.216 |
| Mortality at 1y (among 323 patients) | 75 | 31.5% | 121 | 37.5% | 0.144 |
| Recurrence of bacteremia at 120d from antibiotic termination | 13 | 3.9% | 15 | 3.3% | 0.674 |

Data are depicted as number and percentage or median and Q1-3

^a^Comparison between community and hospital-acquired infection
